# Validation and psychometric evaluation of the Albanian version of the Oral Health Impact Profile-5 (OHIP-5-ALB) in Kosovo

**DOI:** 10.1186/s12903-025-07584-w

**Published:** 2026-01-22

**Authors:** Venera Bimbashi, Asja Čelebić, Robert Ćelić, Besim Hajdari, Mirsad Shkreta, Nikola Petričević

**Affiliations:** 1 Department of Prosthodontics University Dentistry Clinical Centre of Kosovo, Prishtina, Republic of Kosovo; 2 Department of Prosthodontics Alma Mater Europaea, Campus College Rezonanca, Prishtina, Republic of Kosovo; 3 Department of Prosthodontics School of Dental Medicine University of Zagreb, Zagreb, Croatia; 4 Department of Maxillofacial Surgery, Faculty of Medicine School of Dentistry University of Prishtina, Prishtina, Republic of Kosovo; 5 Department of Oral Surgery University Clinical Centre of Kosovo Prishtina, Prishtina, Republic of Kosovo; 6 UBT - Higher Education Institution - Faculty of Dentistry, Pristina, Republic of Kosovo

**Keywords:** OHIP-5-Alb, QHRQoL, Psychometric evaluation, Patient-reported outcome measure, Convergent and discriminant validity, Test-retest reliability

## Abstract

This study validated the Albanian version of the Oral Health Impact Profile-5 (OHIP-5-Alb), a brief, patient-reported outcome measure assessing oral health-related quality of life. Although the OHIP-49 is comprehensive, its length limits practical use, leading to shorter versions like the OHIP-14 and finally OHIP-5, which have been recommended for wide usage.

**Material and Methods**

The OHIP-5-Alb was translated and culturally adapted following rigorous international guidelines and tested across diverse samples: general population (*n* = 441) aged 18–90, having different oral status and educational backgrounds. Dental students (*n* = 30) were included for the test–retest reliability, and prosthodontic patients who needed new complete dentures (*n* = 38) for the responsiveness.

**Results**

Showed good internal consistency (Cronbach’s alpha = 0.725) comparable to other international validations. Test–retest reliability over two weeks showed moderate to excellent intraclass correlation coefficients (0.58–0.98) and similar Weighted Kappa correlation coefficients (0.54–0.92), indicating the questionnaire’s stability over time. Convergent validity was confirmed through strong correlations between self-perceived oral health and the OHIP-5 scores. Discriminant validity was confirmed by significant score differences among different dental status groups. Those with natural teeth reported better OHRQoL than fixed-, and finally removable denture wearers, who faced functional and sensory challenges in chewing and food-flavour perception. Educational level also correlated with denture status; more removable dentures existed in lower educated population, as predicted, while high educated individuals had higher percentage of own teeth or fixed partial dentures, reflecting known socio-dental patterns. Complete denture wearers in general population had significantly lower OHIP5 scores than those who came searching for new complete denture treatment. Responsiveness revealed significant improvements in most OHIP-5 items following new complete denture treatment, except for “Less flavour in food,” consistent with previous findings about sensory limitations and palatal coverage.

**Conclusion**

Limitations include potential sampling bias, underrepresentation of low-income subjects, rural, elderly, and institutionalized individuals, reliance on self-reports without clinical verification in general population, and inclusion of only complete denture treatment for responsiveness. Despite these, OHIP-5-Alb proved to be a reliable, valid, and efficient tool for clinical and epidemiological use in Albanian-speaking populations in the Republic of Kosovo, maintaining good psychometric properties while reducing respondent burden.

**Supplementary Information:**

The online version contains supplementary material available at 10.1186/s12903-025-07584-w.

## Introduction

In recent decades, the assessment of therapeutic and rehabilitation outcomes in dental medicine has expanded beyond objective clinical parameters. Increasing emphasis is placed on patients’ subjective experiences, including improvements in oral health-related quality of life (OHRQoL) and overall well-being. The patient’s perception of treatment outcomes is an increasingly important component of evidence-based evaluations. To facilitate a more objective assessment of oral health-related quality of life (OHRQoL) from the patient’s perspective, several multidimensional standardized questionnaires have been developed, including the Geriatric Oral Health Assessment Index (GOHAI) [1], Oral Impacts on Daily Performances (OIDP) [2], and, the most popular instrument, the Oral Health Impact Profile (OHIP) [3]. In addition, unidimensional instruments focusing on specific aspects of oral health have also been developed, such as the Orofacial Aesthetic Scale (OES), which assesses orofacial aesthetics [4–6], and the Chewing Function Questionnaire (CFQ), which evaluates self-perception of chewing performance [7, 8].

Among the multidimensional instruments, the OIDP assesses the negative impacts of oral conditions on ten daily activities, such as eating, speaking, oral hygiene, physical activity, sleep, relaxation, smiling, emotional well-being, and social interactions. It has also been adapted for use in specific age groups [9]. GOHAI is an age-specific OHRQoL instrument developed for the assessment of oral health in older adult populations aged 65 years and older. The OHIP is a validated instrument designed to measure the functional, psychological, and social consequences of oral health problems; it was developed by Slade and Spencer in 1994 [3] and was originally composed of 49 items grouped into seven dimensions. Although it is a comprehensive instrument encompassing a wide range of oral health impacts, its length has been identified as a limitation in clinical and research settings, where time constraints may hinder its practical application. This often resulted in incomplete responses, potentially compromising the quality of the selected data. Therefore, shorter versions have been developed, and 49 questions have been reduced to 14 [10]. The Oral Health Impact Profile-14 (OHIP-14), also originally developed by Slade G.D. in 1997, is a shortened version of the original OHIP-49 [10]; however, it retained the conceptual framework of the OHIP-49 questionnaire. It has been widely used to assess OHRQoL across diverse populations and settings, with translations available in many languages worldwide. A systematic review of the OHRQoL instruments revealed that the OHIP-14 was the most frequently used instrument (26.8%), followed by the GOHAI (14.0%), OHIP-49 (11.7%) and OIDP (9.7%) [9]. Additionally, several specialized OHIP versions have been developed, such as OHIP-Esthetics [11], OHIP-TMD [12, 13], and OHIP-EDENT [14].

After M.T. John reported that the German version of the OHIP comprises only four dimensions of OHRQoL [15], he initiated a comprehensive international research project to investigate whether the four-dimensional structure is consistent across diverse populations and languages. The project titled “The Dimensions of OHRQoL,” representing an international multicounty study, included a large sample of both general population subjects and prosthodontic patients. The results demonstrated that the OHIP can be effectively conceptualized as comprising only four distinct dimensions: oral function, orofacial pain, orofacial appearance, and psychosocial impact [16, 17].

Additionally, to minimize the time burden associated with completing OHIP questionnaires, an ultrashort version, i.e., the OHIP-5, was developed, consisting of only five items, each representing at least one of the four dimensions of the OHRQoL structure [18, 19]. According to the results of the Recommendations Project [20], all short-form versions of the OHIP demonstrated comparable performance, with strong correlations observed among the summary scores of the 5-, 14-, 19-, and 49-item versions (*r* = 0.91–0.98). The conclusion was that a reliable and practical assessment of OHRQoL across diverse settings and oral health conditions can be effectively conducted via the shortest 5-item OHIP version [20]. Furthermore, Reissmann highlighted the OHIP-5 as the most promising tool for assessing OHRQoL in adult subjects across various clinical and nonclinical environments [21].

In the Albanian language spoken in Kosovo, longer versions of the OHIP questionnaire, such as the OHIP-49 and OHIP-14, have already been validated [22, 23]. Given the growing trend of assessing OHRQoL and treatment outcomes via the ultrashort OHIP-5 questionnaire, particularly in large-scale international studies, the need to develop the Albanian version of the OHIP-5 in the Republic of Kosovo has become evident. Therefore, this study aimed to translate and validate the OHIP-5 instrument for the Albanian-speaking population in the Republic of Kosovo.

## Materials and methods

### Translation and back translation of the OHIP5 questionnaire into the Albanian language spoken in the Republic of Kosovo

The translation of the OHIP-5 into the Albanian language followed a standardized forward–backward translation procedure. Initially, the English version was independently translated into Albanian by a team comprising a professional translator, an Albanian-speaking resident living in the United States, and a dental professional with five years of clinical experience in an English-speaking country and strong proficiency in English. Semantic similarity was carefully assessed during the forward–backward translation process. Discrepancies in wording or significance were reviewed by an expert committee composed of bilingual dental professionals and linguists. Any semantic inconsistencies were resolved through discussion and consensus to ensure that the Albanian version retained the same conceptual meaning as the original OHIP5. This preliminary version in the Albanian language was then reviewed for clarity and cultural appropriateness by a group of dental professionals and patients. Following their approval, the Albanian version underwent back-translation into English. This was carried out by a dentist originally trained in Kosovo, currently practicing in the United Kingdom after diploma recognition, in collaboration with a second professional translator.

Finally, the back-translated version was compared with the original English questionnaire by two native English-speaking dental professionals and five native English-speaking dental patients residing in the UK. Through a consensus-based review, they confirmed that the back-translated items accurately reflected the meaning of the original, indicating no substantial semantic discrepancies. Furthermore, the items of the final version of the OHIP-5-Alb were also compared to the same items of the already translated and validated version of the OHIP-Alb-49 [22] to ensure that no differences exist between the two versions.

The English version and the translation of the OHIP-5 into the Albanian language spoken in Kosovo are presented in the Appendix.

### Participants, ethics approval and consent to participate

The participants in this study were recruited through random sampling from the general population, consecutive sampling among individuals seeking dental care, and targeted selection of dental students. Participants were recruited between October 2024 and June 2025. Data collection, including the test–retest assessment, was completed over a period of approximately 9 months. The sample size was determined according to recommendations for validation studies, i.e. Consensus-based Standards for the selection of health Measurement Instruments (COSMIN), which suggest a minimum of 5–20 participants per item (ranging from 25 to 100 participants for a 5-item scale) [24]. Considering that, and to allow for subgroup analyses, a total of 441 participants were enrolled, surpassing the recommended minimum. Accordingly, the sample is considered a convenience sample. Individuals who were not native Albanian speakers in Kosovo or who had cognitive difficulties were not eligible to participate. The inclusion criteria were willingness to participate and complete a brief questionnaire, age over 18 years, and basic literacy.

The study received approval from the Ethics Committee of the University Dentistry Clinical Center of Kosovo No. 385/2–2025. Every participant was provided with a detailed explanation outlining the study’s objectives, procedures, and assurances of confidentiality. The study was conducted in full accordance with the ethical principles outlined in the Declaration of Helsinki. Data collection was carried out among a heterogeneous group of participants in the general population, including subjects accompanying patients in several dental clinics, vendors selling fruits and vegetables at local markets (to ensure representation of the rural population), bank employees, postgraduate students from the Faculties of Pharmacy and Engineering, staff members working in a pharmaceutical manufacturing facility in one primary school, and municipal workers engaged in cleaning and maintenance tasks. A consecutive sample of dental medicine students was included in the test–retest reliability assessment on a voluntary basis. Additionally, patients requiring new complete dentures were consecutively recruited to assess the instrument’s responsiveness on a voluntary basis.

The participants were provided with a single-sheet-printed version of the OHIP5-Alb questionnaire, accompanied by several additional items. These supplementary questions gathered demographic information (age and gender), fixed or removable denture usage (yes or no), and a self-assessment of their oral health on a five-point Likert scale (1 = completely unsatisfied to 5 = completely satisfied). Each item of the OHIP5-Alb was also rated via a five-point Likert scale, which assesses negative experiences over the past seven days: 0 = never, 1 = hardly ever, 2 = occasionally, 3 = fairly often, and 4 = very often [25]. Table 1 presents participants included in the psychometric validation of the OHIP5-Alb, characterized by study objectives, the proportion of female respondents, their mean age with corresponding standard deviations, and the sampling strategy.

**Table 1:** Characteristics of the participants included in the psychometric evaluation of the OHIP-5 Alb, including research purpose, percentage of female respondents, mean age with standard deviations, and oral status of the participating subjects

Sample	Number of participants	Females	Age (years)	Oral Status (*N*), (%)	Sampling	Research purpose
			Mean ± SD			
General population	441	49.0%	45.9 ± 19.05	Natural teeth (194), (44%)	random	internal consistency convergent validitydivergent validity
				Fixed partial denture (35), (7%)		
				Complete dentures (143,) (32.4%)		
				Partial removable denture (73), (16.6%)		
Patients with a treatment demand (new conventional removable dentures)	38	26.3%	62.89 ± 8.99	Old conventional removable dentures (38), (100%)	consecutive	responsiveness
Students	30	63.3%	23.57 ± 1.01	Natural teeth (30), (100%)	consecutive	test-retest reliability

*SD* standard deviation, *N* number

### Reliability

Two aspects of reliability were evaluated: internal consistency and test–retest reliability. Internal consistency was evaluated in the general population (Table 1) by computing Cronbach’s alpha coefficient, which reflects the degree of correlation among all possible combinations of items. The alpha coefficients when one of the items was excluded, as well as the mean interitem correlation for the OHIP scores, were also calculated. Additionally, Guttman’s split-half reliability coefficient was also calculated. Values exceeding 0.7 were considered indicative of acceptable reliability measures, whereas interitem correlations exceeding 0.20 were considered satisfactory [26].

Test-retest reliability was evaluated via a convenience sample of 30 dental students (see Table 1). The OHIP-5 Alb questionnaire was administered twice, with a two-week interval between sessions. The participants were instructed to report if they noticed any change in oral health during the 15-day period (any changes in oral health conditions would result in exclusion). Intraclass correlation coefficients (ICCs) were determined via one-way repeated-measures ANOVA. Values exceeding 0.8 were interpreted as indicating excellent reliability, whereas those between 0.6 and 0.8 indicated good reliability [27]. Since the OHIP-5 items were measured on an ordinal scale, we additionally calculated weighted Cohen’s Kappa coefficients to assess the test–retest agreement for individual items, and Spearman’s rank correlation coefficient (ρ) for the total OHIP-5 summary scores. Kappa coefficients from 0 to 0.2 were considered low, 0.2–0.4 fair, 0.4–0.6 moderate (acceptable), 0.6–0.8 substantial, and 0.8–1.8 excellent, respectively [28]. The Wilcoxon signed-rank test was used to evaluate the significance of differences between the two measurement sessions.

### Validity

Two aspects of validity were assessed for the OHIP-5 Alb questionnaire: convergent validity and discriminant (known-groups) validity. Convergent validity was evaluated by comparing participants’ responses to the OHIP-5 to a single global item assessing self-rated oral health: “How would you rate your overall oral and dental health?” Responses to a single question were given on a 5-point Likert scale (1 = poor to 5 = excellent). The association between the OHIP-5-Alb summary score and the self-assessment of oral health was examined via Spearman’s rank correlation.

To assess discriminative validity, participants were grouped according to their prosthodontic status: no dentures, fixed partial dentures, or removable dentures (partial or complete). It was hypothesized that individuals with natural dentition would report the best oral health-related quality of life (OHRQoL), followed by those with fixed prostheses and those with removable dentures. Differences among the groups were analyzed via one-way ANOVA, with Scheffé post-hoc tests applied to determine statistically significant differences. It was also hypothesized that individuals with lower levels of education would have a greater prevalence of removable dentures than those with university or postgraduate degrees. This association was evaluated via chi-square (χ²) tests to compare frequencies across educational groups. Moreover, we also hypothesized that individuals from the general population with complete dentures (CD) would have lower OHIP-5 summary scores compared to those visiting a dental clinic for treatment with newly manufactured CDs. We compared the two groups using the Mann-Whitney U test.

### Responsiveness

Responsiveness is the ability of a questionnaire to detect significant changes in a participant’s condition or experience over time, whether due to improvements from interventions such as therapy or rehabilitation or declines resulting from natural progression. It reflects the instrument’s sensitivity to change over time. Therefore, it was reasonable to test the responsiveness of the Albanian 5-Item OHIP questionnaire. A convenience sample of 38 patients requiring treatment with new complete dentures was recruited (Table 1). On a voluntary basis, participants agreed to complete the questionnaire twice: once before the therapy and again one month after receiving new removable complete dentures, following all necessary postdelivery adjustments. It was anticipated that OHRQoL impairment would be reduced one month after treatment with new complete dentures and the corresponding adaptation period. To determine the effect of the treatment, the standardized effect size was calculated via the following formula: *(Baseline OHIP-5-Alb score – Follow-up OHIP-5-Alb score)/Standard deviation of the baseline OHIP-5-Alb score.* An effect size of 0.2 was considered small, 0.5 was considered moderate, and 0.8 was considered large [29].

## Results

The general population sample included 441 participants. The demographic and clinical characteristics of the participants, such as sex distribution, mean age with standard deviation, oral status (no prosthesis, fixed partial dentures, complete removable dentures, and partial removable dentures), and study purpose are summarized in Table 1. The participants’ ages ranged from 18 to 90 years. Table 1 also presents the same characteristics for the dental students (*n* = 30) who participated in the test-retest reliability assessment, as well as for the patients with very old removable dentures requiring treatment, who received new removable dentures and were included in the responsiveness evaluation (*n* = 38).

Regarding the educational background of the participants, 8.6% (*n* = 38) had completed only primary education, which consisted of four years for older participants and eight years for those educated after World War II. A total of 40.1% (*n* = 177) had completed secondary education, whereas 45.1% (*n* = 199) held a university degree (bachelor’s or master’s). Additionally, 6.1% (*n* = 27) had completed postgraduate studies.

### Reliability

The Cronbach’s α coefficient was used to assess internal consistency in a sample of the general population consisting of 441 subjects aged 18–90 years. Table 1 shows their mean age and sex distributions. The overall alpha coefficient of 0.725 indicated satisfactory reliability, whereas the removal of any single item produced α values ranging from 0.57 to 0.72 (Table 2). Guttman’s split half coefficient was also satisfactory, with a value of 0.708. Table 2 also lists mean scores and standard deviations for each OHIP-5 Alb item. The interitem correlation matrix (Table 3) revealed that every correlation surpassed the 0.20 threshold.

**Table 2:** Mean values of each item, standard deviations and Cronbach alpha coefficients after one item of the OHIP-5 Alb questionnaire was deleted in the general population sample

OHIP-5 Alb			
5-Item OHIP Questionnaire	Mean	Standard Deviation	Cronbach alpha if Item Deleted
Difficulty chewing	0.98	0.94	0.57
Painful aching	0.51	0.71	0.72
Uncomfortable with appearance	1.09	1.05	0.67
Less flavour in food	1.12	1.16	0.72
Difficulty doing usual jobs	0.37	0.73	0.70

**Table 3:** Inter-Item correlation matrix for the Albanian OHIP-5 version (General population)

Difficulty chewing	1.0				
Painful aching	0.47	1.0			
Uncomfortable about appearance	0.50	0.27	1.0		
Less flavour in food	0.56	0.44	0.36	1.0	
Difficulty doing usual jobs	0.41	0.45	0.30	0.20	1.0

Test–retest reliability was evaluated as another measure of reliability. A convenience sample of 30 consecutive dental students, who were dentate and willing to participate, was selected. The mean age and sex distributions of the participants are presented in Table 1. To ensure consistency, participants were required to maintain stable oral and dental conditions throughout the two-week interval between the two administrations of the same questionnaire. Participants who reported an experience of any changes in their oral or dental condition during the interval had to be excluded from the analysis. Table 4 presents the results of the test–retest reliability analysis of dental students who completed the OHIP-5 questionnaire twice over a two-week interval, as evaluated via one-way repeated-measures ANOVA. No statistically significant differences were detected for individual item scores or summary scores across the two administrations (*P* > 0.05). The ICC values were also calculated and demonstrated good to excellent reliability, as well as weighted Kappa coefficients for individual items or Spearman’s rho for the summary scores, as additional measures of agreement [28].

**Table 4:** Test–retest reliability analysis in 30 dental students who completed the OHIP-5 questionnaire twice over a two-week interval, evaluated using one-way repeated-measures ANOVA, as well as descriptive statistics, weighted kappa correlation for individual items, spearman’s Rho correlation coefficient for the summary scores; significance of the differences tested by Wilcoxon sign rank test

Ohip 5 Items	Mean scores	SD	Median	Interquartile range	ICC	Weighted Kappa	Z	*P*
Difficulty chewing 1	0.50	0.78	0	1	0.76	0.88	−1.42	0.16 N.S.
Difficulty chewing 2 (retest)	0.37	0.56	0	1				
Painful aching	0.30	0.53	0	1	0.96	0.92	−1.00	0.32 N.S.
Painful aching 2 (retest)	0.33	0.61	0	1				
Uncomfortable about appearance 1	0.43	0.802	0	1	0.93	0.80	−0.57	0.56 N.S.
Uncomfortable about appearance 2 (retest)	0.47	0.860	0	1				
Less flavour in food	0.17	0.590	0	0	0.98	0.84	−1.00	0.32 N.S.
Less flavour in food 2 (retest)	0.13	0.430	0	0				
Difficulty doing usual jobs 1	0.03	0.18	0	0	0.58	0.54	−1.44	0.16 N.S.
Difficulty doing usual jobs 2 (retest)	0.10	0.300	0	0				
Ohip 5 Summary score 1	1.43	2.21	1	2	0.97	§	−0.38	0.71 N.S.
Ohip 5 Summary score 2 (retest)	1.40	2.19	1	2				

§ = spearman’s Rho = 0.93; SD = standard deviation; *P* = *p* value; N.S.= not significant

### Validity

#### Known groups validity

Significant differences in OHIP-5 Alb summary scores were observed among participants with natural dentition (NT), FPDs, CDs, and RPDs, as determined by one-way ANOVA and Scheffé post hoc analyses. Notably, the item *“difficulty chewing”* showed significant differences across groups, except between the two types of removable denture wearers, who reported similar challenges with mastication. For the items *“Painful aching”* and *“Feeling uncomfortable about appearance”*, only individuals with natural teeth differed significantly from those in the other groups. As hypothesized, participants with removable dentures, both partial and complete, reported significantly more negative impacts than those with natural teeth or fixed prostheses did for the items *“less flavor in food”* and *“difficulty performing usual tasks”* (Table 5).

**Table 5:** Spearman’s rank correlation coefficients between self-rated oral health and individual items of the OHIP-5-Alb, including the total summary score (general population sample)

Self-assessed oral health	Difficulty chewing	Painful aching	Uncomfortable about appearance	Less flavour in food	Difficulty doing usual jobs	OHIP 5 Alb Summary score
Spearman’s rho	−0.683**	−0.505**	−0.602**	−0.533**	−0.544**	−0.795**
Significance	< 0.001	< 0.001	< 0.001	< 0.001	< 0.001	< 0.001
Number of participants	441	441	441	441	441	441

** *P* < 0.001

**Fig. 1:**
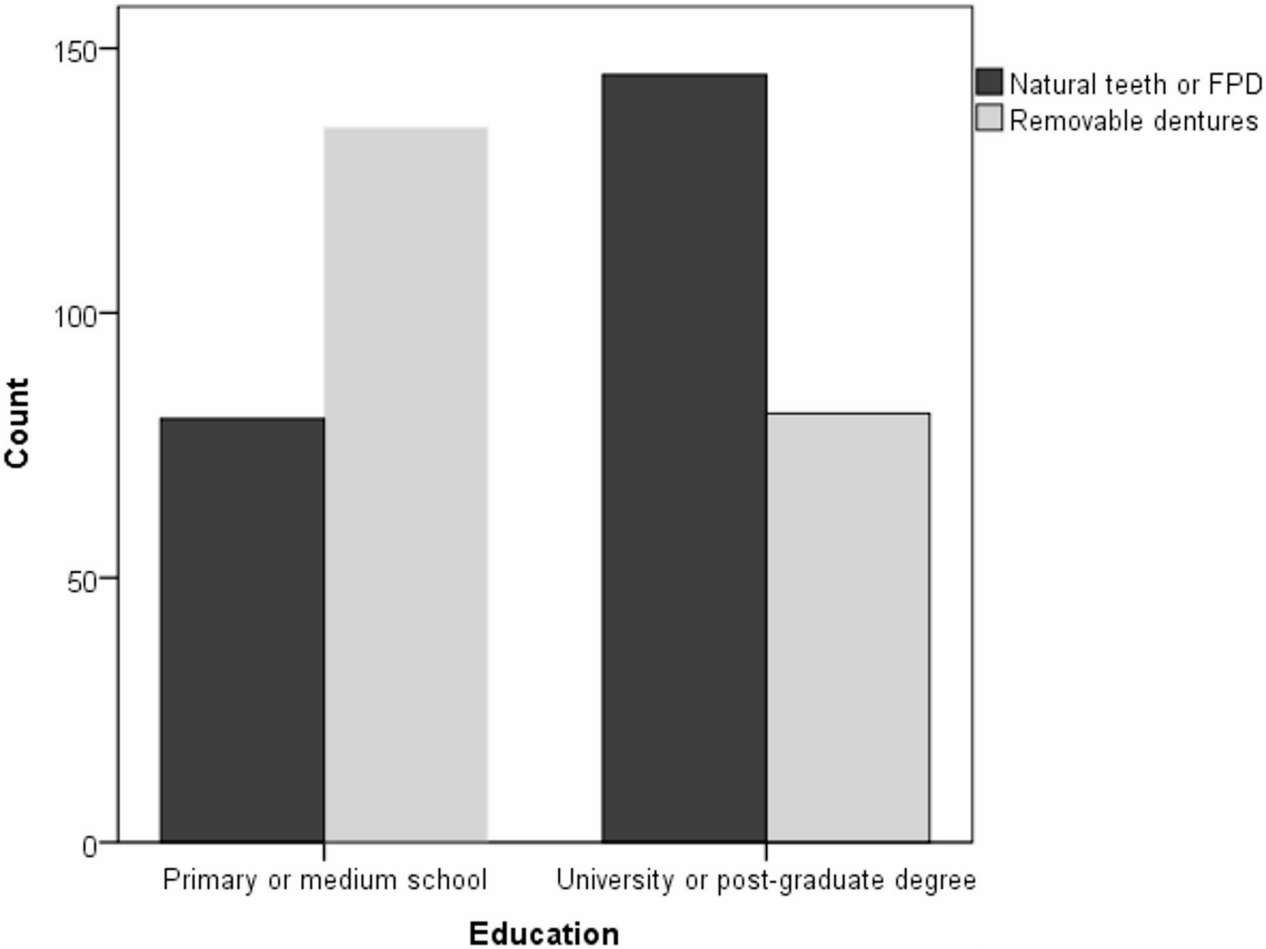
Frequency of natural teeth or fixed partial dentures versus removable dentures (including both complete and partial) depending on the educational level

An additional analysis of discriminant validity assessed the distribution of removable dentures across varying educational levels via the chi-square (χ²) test. As expected, individuals with lower educational levels had a significantly greater prevalence of removable dentures, whereas those with university or postgraduate degrees more commonly retained natural dentition or had fixed partial dentures (χ² = 32.03, *P* < 0.001) (Fig. 1).

Individuals from general population wearing CDs had significantly lower OHIP-5 summary scores compared to individuals visiting a dental professional for a treatment with new CDs (Z = 5.57; *P* < 0.001) (Table 6).

**Table 6:** One-way ANOVA with Scheffé post-hoc tests for assessing discriminant validity: comparison of OHIP-5-Alb scores among participants with natural dentition, fixed partial dentures, complete removable dentures, and partial removable dentures in the general population (Degrees of Freedom = 3; 437; 440)

OHIP-5 Item	Oral Status	*N*	Mean Score	Standard Deviation	F	*p*		Sheffe post-hoc			
								NT	FDP	CD	RPD
Difficulty chewing	Natural teeth	194	0.26	0.50	148.66	< 0.001	NT		*	*	*
	FPD	31	0.84	0.58			FPD	*		*	*
	CD	143	1.63	0.86			CD	*	*		
	RPD	73	1.68	0.64			RPD	*	*		
								NT	FDP	CD	RPD
Painful aching	Natural teeth	194	0.35	0.58	8.70	< 0.001	NT		*	*	*
	FPD	31	0.83	0.45			FPD	*			
	CD	143	0.52	0.79			CD	*			
	RPD	73	0.75	0.86			RPD	*			
								NT	FDP	CD	RPD
Uncomfortable about appearance	Natural teeth	194	0.56	0.93	38.88	< 0.001	NT		*	*	*
	FPD	31	1.45	0.85			FPD	*			
	CD	143	1.38	0.97			CD	*			
	RPD	73	1.75	0.92			RPD	*			
								NT	FDP	CD	RPD
Less Flavour in food	Natural teeth	194	0.23	0.80	211.52	< 0.001	NT			*	*
	FPD	31		0.55			FPD				
	CD	143	2.02	0.72			CD	*			
	RPD	73	2.08	0.78			RPD	*			
								NT	FDP	CD	RPD
Difficulty doing usual jobs	Natural teeth	194	0.19	0.52	8.75	< 0.001	NT			*	*
	FPD	31	0.32	0.60			FPD			*	*
	CD	143	0.52	0.95			CD	*			
	RPD	73	0.59	0.64			RPD	*			
								NT	FDP	CD	RPD
Ohip-5 Summary score	Natural teeth	194	1.52	1.78	166.80	< 0.001	NT		*	*	
	FPD	31	3.94	1.26			FPD	*		*	*
	CD	143	6.15	2.86			CD	*	*		
	RPD	73	7.04	2.30			RPD	*	*		*

*NT* natural teeth, *FPD* fixed partial dentures, *CD* removable complete dentures, *RPD* removable partial dentures

* asterix present significant differences between groups

### Responsiveness

To assess whether the summary score or individual items of the OHIP-5 Alb are sensitive to changes elicited by the treatment (new complete dentures) performed by a prosthodontic specialist, responsiveness was evaluated (Table 7). To determine significant changes in OHIP-5 scores from baseline to follow-up, the Wilcoxon signed-rank test, a nonparametric method, was applied, followed by calculation of the effect size of a treatment.

**Table 7:** Descriptive statistics of the OHIP5 Alb summary scores for complete denture wearers from general population and those visiting a specialist in prosthodontics for manufacture of new complete dentures and significance of the differences assessed by Mann Whitney U test test

Individuals visiting a specialist in prosthodontics for treatment with new complete dentures (*n* = 38)							
Items	Mean	Std. Deviation	Minimum	Maximum	Median	Interquartile range	Mean Rank
OHIP5 Summary score	9.08	2.56	40	14	9.0	3	130.51 §

Individuals from General population wearing complete dentures (*n* = 143)							
OHIP5 Summary score	6.01	2.79	1	14	5	4	78.37 §

§ = (Z = 5.57; *P* < 0.001)

The effect sizes for the *summary score* and the items *difficulty chewing*,* painful aching*,* and uncomfortable appearance* were large, indicating substantial changes following treatment and statistically significant improvement after treatment. The item *Difficulty Doing Usual Jobs* had a moderate effect size, reflecting a noticeable but less pronounced improvement. In contrast, only the item *Less Flavor in Food* demonstrated a very small effect size and did not exhibit statistically significant changes after treatment (*P* > 0.05).

### Convergent validity

The results of the convergent validity analysis for the Albanian version of the OHIP-5 are shown in Table 8. A significant negative correlation was observed between the self-rated global oral health item and both the individual OHIP-5 items and the overall summary score, as determined by Spearman’s rank correlation coefficient.

**Table 8:** Mean scores and standard deviations with the significance of changes between baseline and post-treatment assessments in 38 patients with a treatment demand (Wilcoxon signed-rank test), accompanied by the calculation of treatment effect sizes

OHIP-5 Alb Items	Mean score	Standard deviation	Z	*P*		Effect size
Difficulty chewing (before treatment)	3.05	0.62	−5.48	< 0.001	2.76	
Difficulty chewing (after treatment)	1.34	0.48				
Painful aching (before treatment)	1.34	0.99	−4.13	< 0.001	0.98	
Painful aching (after treatment)	0.37	0.49				
Uncomfortable about appearance (before treatment)	2.11	0.98	−4.55	< 0.001	1.26	
Uncomfortable about appearance (after treatment)	0.87	0.47				
Less flavour in food (before treatment)	1.16	1.13	−0.90	0.37 (N.S.)	0.18	
Less flavour in food (after treatment)	0.97	0.72				
Difficulty doing usual jobs (before treatment)	1.47	1.08	−2.84	< 0.05	0.63	
Difficulty doing usual jobs (after treatment)	0.79	0.53				
Ohip 5 Summary score (before treatment)	9.18	2.63	−5.02	< 0.001	1.84	
Ohip 5 Summary score (after treatment)	4.34	0.88				

Wilcoxon Signed Ranks Test, Based on positive ranks

## Discussion

Dental medicine increasingly recognizes patient-reported outcome measures (d-PROMs), especially OHRQoL. Standardized questionnaires such as the OHIP have become essential tools. Longer OHIP versions offer comprehensive evaluation but require considerable time; hence, shorter tools like the ultrashort OHIP-5 are increasingly favored, especially nowadays, when time is precious. When developed, the OHIP5 demonstrated strong correlations with the longer versions. It minimizes respondents’ burden, but still encompasses key dimensions of oral function, pain, appearance, and psychosocial impact [19–21], and has been successfully validated across diverse cultural settings [18–20, 30–40]. Given the increasing international use and the lack of a validated version in the Albanian language spoken in Kosovo, the validation of this instrument was considered essential. The rigorous worldwide accepted translation and cultural adaptation of the OHIP-5 into the Albanian language was performed. The diverse and representative sampling strategy, including the general population, dental students, and prosthodontic patients, strengthens the validation process by assessing multiple psychometric properties, including internal consistency, test-retest reliability, and responsiveness. The general population sample (*n* = 441) encompassed a broad age range (18–90 years), and diverse oral statuses. The educational distribution also reflects a heterogeneous sample, increasing the robustness of the instrument across socio-educational levels. The main finding of this study is that the OHIP-5-Alb demonstrated good psychometric properties, supporting its use in large-scale studies where time for assessment is limited, thus supporting its applications in meaningful international comparisons.

### Reliability

As a measure of internal consistency, the Cronbach’s alpha coefficient of 0.725 calculated in the general population sample falls within the acceptable range (≥ 0.70). Comparable results have been reported in other studies, such as English (0.75) [19], Arabic (0.78) [33], Macedonian (0.756) [35], Swedish (0.77) [36], German (0.72) [18], Croatian (0.702), Slovenian (0.766) [38], and Serbian (0.784) [40] OHIP-5 validations, whereas Cronbach’s alpha values were slightly higher in the Chinese 0.868 [34], Japanese (0.81) [30], Persian (0.85) [31], and Spanish (0.83) [32] versions. Thai version of the OHIP5 questionnaire assessed correlation between the OHIP5 and OHIP14 or OHIP49 [39].

Since Cronbach’s alpha is influenced by the number of items in a scale, the longer versions of the Albanian OHIP questionnaires (OHIP-49 and OHIP-14) understandably had higher alpha values (0.94 and 0.86, respectively) [22, 23]. An alpha value > 0.90 might even suggest items’ redundancy, as items may be too similar. When an item was deleted, all the alpha values were reduced by a small amount (0.57–0.72), demonstrating that each item contributed positively to the scale’s reliability. However, the item ‘Difficulty chewing’ showed the lowest value when it was deleted (0.57), indicating that removing it would reduce the overall reliability more than removing any other item, thus underlining its importance for maintaining internal consistency. The split-half reliability measures the correlation between two halves of the test (odd vs. even items) and adjusts it to estimate the full-scale reliability. It was used as a supporting method and showed acceptable values.

Test–retest reliability was assessed to evaluate the temporal stability of the OHIP-5-Alb instrument over a two-week interval in a homogenous sample of 30 dentate dental students with stable oral health conditions Dental students were selected because it was assumed that any changes in oral health conditions occurring during the observation period would be more readily noticed by them compared to participants from the general population. However, dental students were younger and more homogeneous in educational background than the general population sample used for internal consistency analysis. This difference may have contributed to slightly higher reliability estimates due to better understanding of questionnaire items and reduced variability. Future studies should evaluate test–retest reliability in more demographically diverse samples to enhance generalizability. The results revealed no statistically significant differences between the initial and repeated administrations for any of the five OHIP-5 items or the summary score (Table 4; *p* > 0.05). Furthermore, the intraclass correlation coefficients (ICCs) ranged from 0.58 to 0.98, demonstrating moderate to excellent reliability. Because OHIP-5 scores were skewed and ordinal in nature, the use of ICC for test–retest reliability has certain limitations. Nevertheless, this approach was chosen to maintain comparability with prior OHIP-5 validation studies. However, we additionally calculated weighted Cohen’s Kappa coefficients to assess the test–retest agreement for individual items, and Spearman’s rank correlation coefficient (rho) for the OHIP-5 summary scores (Table 4). All agreements were almost perfect except for the Item: *‘Difficulty doing usual jobs’* which showed moderate agreement [28]. More variability was present in participants’ interpretation or recall of this question. Further validation studies involving larger and more diverse populations are warranted to confirm the stability of this item and to strengthen the reliability of the OHIP-5-ALB. Our findings are consistent with previous validation studies of the OHIP-5 in other languages and cultural contexts [18–20, 30–40], supporting the reproducibility and measurement stability of the Albanian version of the OHIP-5 for use in longitudinal and population-based research. Future research may benefit from introducing more diverse samples from general population for test-retest reliability.

### Validity

The findings support the validity of the Albanian OHIP-5 through both convergent and discriminant validity analyses. Convergent validity was demonstrated by strong, statistically significant negative correlations between self-perceived oral health and each OHIP-5 item, as well as the summary score (ρ = − 0.795, *p* < 0.001). This suggests that individuals who rated their oral health more negatively also reported greater impacts on their quality of life, which aligns with the expected theoretical constructs. The negative correlation sign is attributed to the opposing score directions: in the self-rated oral health item, higher scores indicated better self-perceived oral health (on a scale from 1 to 5), whereas in the OHIP-5, lower scores denoted fewer oral health problems and thus better OHRQoL.

Discriminant validity was confirmed through significant differences in the OHIP-5 summary and item scores across groups with different dental/prosthodontic statuses in the oral cavity. As hypothesized, the best OHRQoL was found in subjects with their own dentition, followed by those with FPDs, CDs, and RPDs. Compared with those with natural teeth or fixed prostheses, individuals with removable dentures (CDs and RPDs) reported significantly worse outcomes, especially with respect to chewing difficulty, food flavor, and difficulty performing usual tasks. These differences reflect the known functional limitations and psychological impacts associated with removable prosthodontics. The items “difficulty chewing” and “less flavor in food” were particularly not sensitive enough to distinguish between complete and partial denture wearers, who reported similar limitations, which is logically attributed to palatal coverage and the potential instability or movements of the denture base during mastication, in both types of removable dentures [41–45]. Moreover, removable dentures present a potential risk factor for a poor ingestible food profile or reduced sensory input and even cognitive decline over time [46–48]. Conventional removable denture wearers are often forced to use denture adhesives [49].

Further evidence of discriminant validity came from the observed association between educational level and dental/denture status in the oral cavity, where individuals with lower education more frequently had removable dentures (χ² = 32.03, *p* < 0.001) as predicted, as it is a pattern consistent with broader socio-dental literature [50, 51], especially in very old adults [52–55]. Populations having low socioeconomic status or living outside a major city have also been associated not only with a high prevalence of edentulism and removable dentures but also with reduced denture replacements when necessary [56]. As predicted, CD wearers from general population had lower OHIP5 Alb summary scores than those who perceived treatment needs and came to a dental office for a new CD manufacture. However, future studies should focus on OHRQoL among individuals with dentures of varying age and quality, which would provide clinically meaningful insights, and will be the subject of future study.

All validity findings (convergent and discriminant) in this study confirm that the OHIP-5-Alb has valid measurement properties, making it appropriate for use in population studies and clinical assessments within Albanian-speaking populations.

### Responsiveness

The responsiveness analysis of the OHIP-5 Alb demonstrated that the instrument is sensitive to clinically meaningful changes following prosthodontic treatment, in this case, the provision of new complete dentures. Statistically significant improvements were observed in most items and the overall summary score. It is very important to measure the responsiveness and effect sizes of a treatment, as objective clinical parameters and patients’ perceptions can differ, especially regarding removable denture rehabilitation [57–61]. The largest effect sizes (improvements) were recorded for the items *Difficulty chewing*,* Discomfort about appearance*, and *Painful aching*, as well as for *the summary score.* In contrast, the item *Less Flavor in Food* demonstrated a very small effect size, as a logical consequence of palatal, as well as other denture-bearing areas coverage. The findings indicate that although most aspects of oral health-related quality of life were significantly improved with new prostheses, certain areas such as the sensory perception of food flavor were less responsive to the intervention, consistent with previous studies [62, 63]. New conventional removable dentures led to increased salivary flow but not to increased food flavor perception [62] or tactile sensory perception in the denture-bearing area [63].

### Limitations of the study

Although this study aimed to include a diverse cross-section of the general population (greenery vendors at local markets were included to represent rural residents), it is likely that individuals with the lowest income or those living in remote rural areas were underrepresented. Older adults residing in care facilities or those with limited mobility and those who needed assistance were not included. Additionally, all the data were collected through self-reported measures, and the use of fixed and removable dentures was not clinically verified. Very elderly individuals and those with severe oral or systemic health conditions are likely underrepresented in the general population. The test–retest reliability group comprising of dental students represented a convenience sample with predominantly healthy natural dentition, excluding peers of similar age with different socioeconomic backgrounds or different oral health behaviors. Thus, sampling bias cannot be entirely excluded. Furthermore, the responsiveness analysis included only treatment with new complete dentures, whereas long-term interventions such as implant-supported prosthodontics or orthodontic therapy were not included due to their extended treatment durations [64–69]. In the subgroup of removable denture wearers, women were underrepresented (26.3%). This gender imbalance may limit the generalizability of findings and partly reflect social, cultural, or healthcare access differences in the examined population from Kosovo. Future studies should ensure a more balanced gender distribution to confirm the stability of psychometric properties across sexes.

## Conclusion

Despite the study’s limitations, the findings indicate that the OHIP-5 Alb questionnaire demonstrates acceptable psychometric properties, such as reliability, validity, and responsiveness. Its brevity and acceptable psychometric properties suggest it may be a practical option for both clinical and research settings, particularly where time constraints or large sample sizes require a short, efficient instrument. The OHIP-5 Alb also offers a useful tool for assessing OHRQoL in Albanian-speaking populations, supporting its potential role in international and clinical applications.

## Supplementary Information

Supplementary Material 1.

## Data Availability

The records supporting the findings of this study are obtainable from the corresponding author upon reasonable request.

## Abbreviations

OHIP-5 Alb: Oral Health Impact Profile 5 in Albanian
OHRQoL: Oral Health Related to Quality of Life
GOHAI: Geriatric Oral Health Assessment Index
OIDP: Oral Impacts on Daily Performances
OES: Orofacial Aesthetic
CFQ: Chewing Function Questionnaire
NT: Natural dentition
FPDs: Fixed partial dentures
CDs: Complete Dentures
RPDs: Removable Partial Dentures
ICC: Intraclass Correlation Coefficient
