# Supplementary material for: Validation and psychometric evaluation of the Albanian version of the Oral Health Impact Profile-5 (OHIP-5-ALB) in Kosovo
Source: BMC Oral Health. 2026 Jan 22;26:414. doi: 10.1186/s12903-025-07584-w (PMC12952158; doi:10.1186/s12903-025-07584-w)
Supplement: Supplementary file 1 — Supplementary Material 1. [file 12903_2025_7584_MOESM1_ESM.pdf]

OHIP-5 in English / translation in Albanian

**Oral function:**
**Q1. Have you had difficulty chewing any foods because of problems with your teeth, mouth, dentures or jaw?**
**In Albanian**
**Q1.** A ke pasur vështërsi gjatë përtypjes, për shkak të problemeve me dhëmbë, gojë, nofulla apo proteza?

-

**Orofacial pain:**
**Q2. Have you had painful aching in your mouth?**
**In Albanian**
**Q2.** A ke patur dhembje të mëdha në hapsirën e gojës?

**Orofacial appearance:**
**Q3. Have you felt uncomfortable about the appearance of your teeth, mouth, dentures or jaw?**
**In Albanian**
**Q3**. A ke qenë i/e shqetësuar për shkak të problemeve me dhembë, gojë, proteza,nofullë apo proteza?

**Oral function:**
**Q4. Have you felt that there has been less flavour in your food because of problems with your teeth, mouth, dentures or jaws?**
**In Albanian**
**Q4.** A ke pasë pengesa me shije për shkak të problemeve me dhëmbë, gojë, nofullë apo proteza?

**Psychosocial impact:**
**Q5. Have you had difficulty doing your usual jobs because of problems with your teeth, mouth, dentures or jaws?**
**In Albanian**
**Q5**. A të ka ndodhur që të mos mund te punonojsh me kapacitet të plotë për shkak të problemeve me dhëmbë, gojë, nofullë apo proteza?
